# Giant cell arteritis with cervical radiculopathy mimicking polymyalgia rheumatica and elderly-onset rheumatoid arthritis: a case report

**DOI:** 10.1186/s13256-021-03107-7

**Published:** 2021-10-20

**Authors:** Akihiko Nakabayashi, Hiroki Ikai, Yoshinori Katada

**Affiliations:** 1 Department of Nephrology, Diabetology, and Rheumatology, Sakai City Medical Center, Ebaraji-cho 1-1-1, West Ward, Sakai, Osaka 593-8304 Japan; 2 Department of Rheumatology and Allergology, National Hospital Organization Osaka Minami Medical Center, 2-1 Kidohigashi, Kawachinagano, Osaka 586-8521 Japan; 3 Department of Rheumatology, Japan Organization of Occupational Health and Safety Chubu Rousai Hospital, 1-10-6 Koumei Minato-ku, Nagoya, Aichi 586-8521 Japan; 4 Department of Respiratory Medicine and Clinical Immunology, Suita Municipal Hospital, 5-7 Kishibeshinmachi, Suita, Osaka 564-8567 Japan

**Keywords:** Giant cell arteritis, Polymyalgia rheumatica, Elderly-onset rheumatoid arthritis, Cervical radiculopathy, Musculoskeletal ultrasound, Neurological examination

## Abstract

**Background:**

Giant cell arteritis has a wide variety of clinical symptoms, one of them being cervical radiculopathy, which mainly involves the C5 nerve root. If the patient does not develop typical clinical symptoms of giant cell arteritis but has C5 radiculopathy, it may be misdiagnosed as polymyalgia rheumatica or elderly-onset rheumatoid arthritis due to old age, high serum inflammatory markers, and difficulty in raising both upper limbs.

**Case presentation:**

A 72-year-old Japanese man with a month-long history of dyspnea on exertion and with difficulty in raising both upper limbs was referred to our hospital because of elevated serum C-reactive protein (12.62 mg/dL). He had no typical symptoms of giant cell arteritis such as headache, jaw claudication, visual loss, and fever. The patient tested negative for rheumatoid factor and anti-cyclic citrullinated peptide antibody, and matrix metalloproteinase-3 was within the normal range (54.3 ng/mL). Musculoskeletal ultrasound examination showed absence of tenosynovitis, bursitis, and synovitis, and the patient did not meet the classification criteria of polymyalgia rheumatica or rheumatoid arthritis; hence, those two diseases were unlikely. A precise neurological examination suggested bilateral C5 and C6 anterior radiculopathy and left C4 radiculopathy. Since cervical magnetic resonance imaging showed no mechanical causality, cervical radiculopathy of unknown origin was suggested. Fluorodeoxyglucose positron emission tomography/computed tomography revealed increased fluorodeoxyglucose lineal uptake along the vessel walls, including temporal arteries, vertebral arteries, and axillary arteries. Results of the biopsy of the left superficial temporal artery were compatible with giant cell arteritis. He was successfully treated with 30 mg of prednisolone, and both upper limbs could be elevated.

**Conclusions:**

If the patient was misdiagnosed with polymyalgia rheumatica or elderly-onset rheumatoid arthritis based on only clinical symptoms and laboratory data, his symptoms might not improve due to insufficient steroid dose and vascular complications may occur later. Although rare, peripheral neuropathy in giant cell arteritis may include cervical radiculopathy. The musculoskeletal ultrasound and precise neurological examination were the turning points for the diagnosis of this case, and making a careful diagnosis using these methods was important.

**Supplementary Information:**

The online version contains supplementary material available at https://doi.org/10.1186/s13256-021-03107-7.

## Background

Giant cell arteritis (GCA) is a chronic systemic vasculitis that occurs in people older than 50 years and generally affects large- and medium-sized arteries. GCA has a wide variety of symptoms such as headache, jaw claudication, visual loss, and systemic manifestations (anorexia, asthenia, malaise, myalgia, arthralgia, weight loss, and so on), but neurological symptoms may also occur [1]. GCA neuropathy is divided into central and peripheral neuropathy, and peripheral neuropathy occurs at a frequency of 1.1–14% [2–4]. However, radiculopathy is extremely rare in GCA patients with neuropathy [5]. Preferential involvement of the C5 nerve roots is also reported in GCA patients with radiculopathy [5, 6]. Because the deltoid, pectoralis major, and supraspinatus muscles are innervated by the C5 nerve, it is difficult to raise both upper limbs when this nerve is affected. Therefore, if the patient has no typical clinical symptoms of GCA but has C5 radiculopathy, it may be misdiagnosed as polymyalgia rheumatica (PMR) or elderly-onset rheumatoid arthritis (EORA) due to old age, high serum inflammatory markers, and difficulty in raising both upper limbs.

Herein, we report a case of GCA with cervical radiculopathy presenting clinical symptoms similar to those of PMR or EORA, whose diagnostic turning point was musculoskeletal ultrasound examination and precise neurological examination.

## Case presentation

**Fig. 1:**
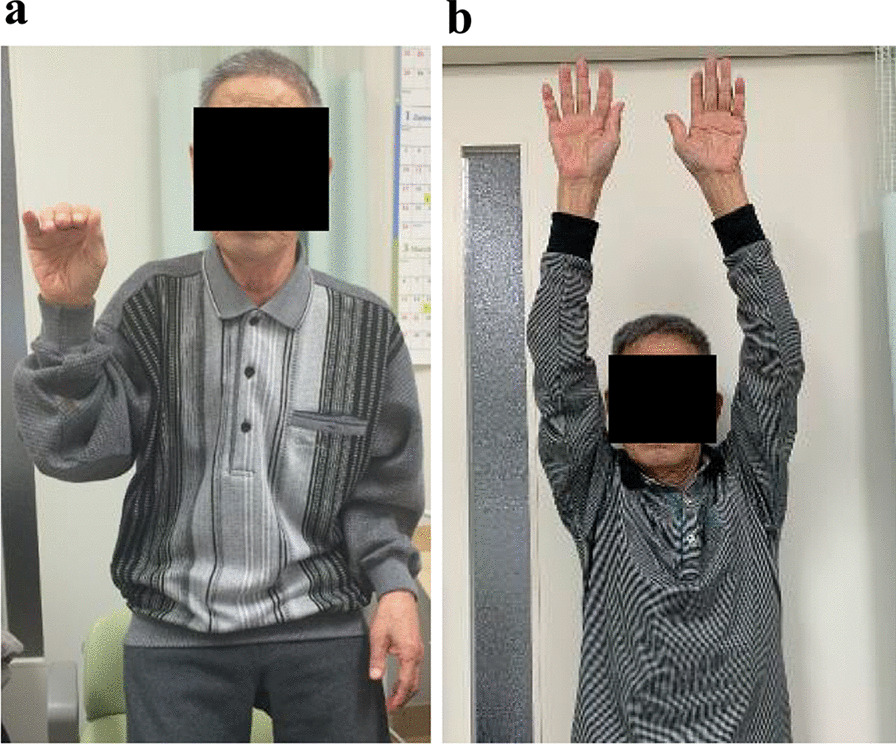
Clinical course of the patient. **a** The upper right limb could not be elevated to the shoulder level, whereas the upper left limb could not be elevated at all before treatment. **b** Both upper limbs could be completely elevated 4 years and 8 months after treatment

**Fig. 2:**
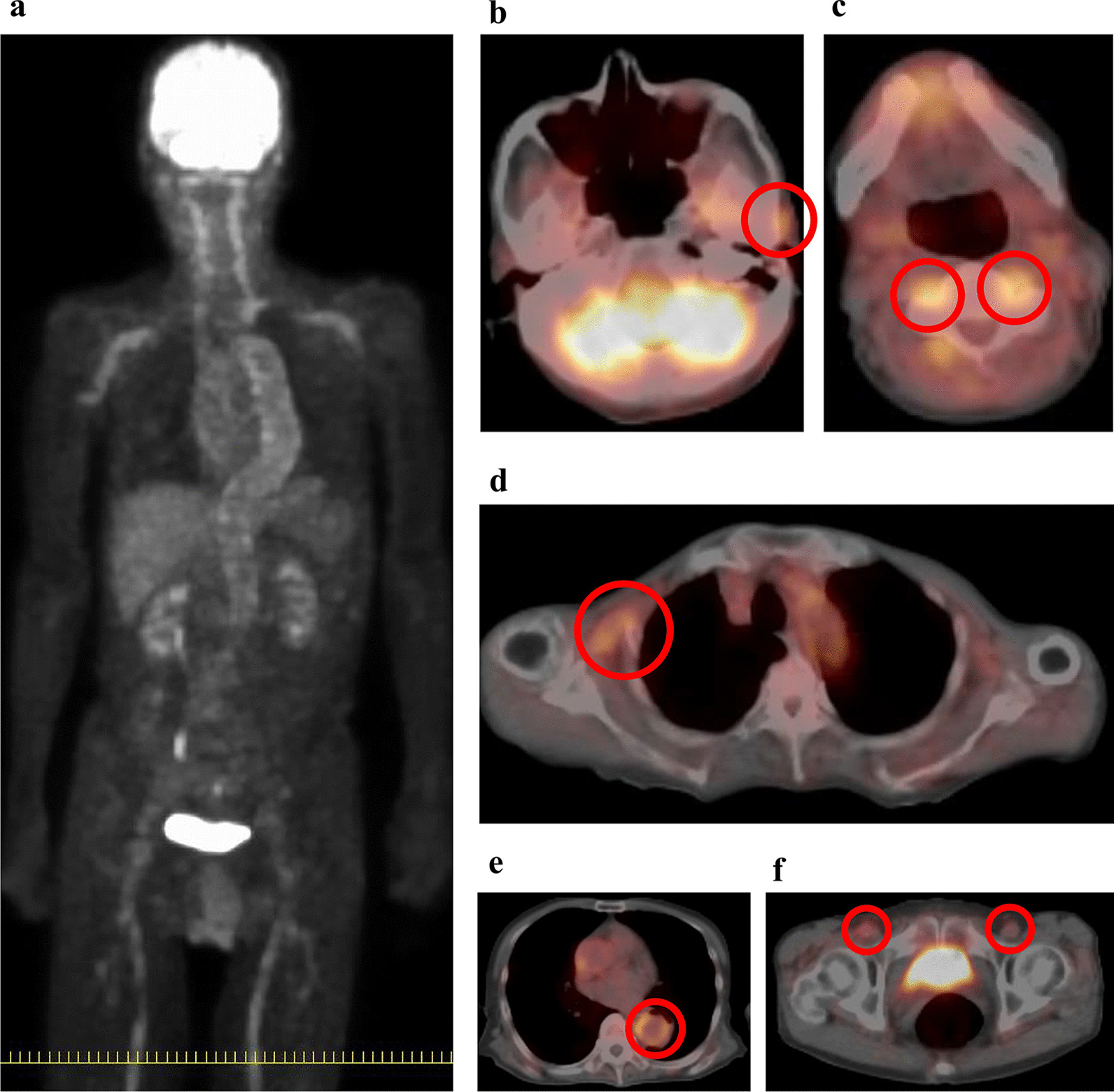
Fluorodeoxyglucose positron emission tomography/computed tomography. **a** Coronal PET image. **b**–**f** Axial PET/CT images. FDG uptake of left temporal artery (**b** circle, SUVmax 3.6), bilateral vertebral arteries (**c** circle, SUVmax 4.4), right axillary artery (**d** circle, SUVmax 3.1), descending aorta (**e** circle, SUVmax 3.8), and bilateral femoral arteries (**f** circle, SUVmax 2.3). PET, positron emission tomography; CT, computed tomography; FDG, fluorodeoxyglucose; SUVmax, maximum standardized uptake value

**Fig. 3:**
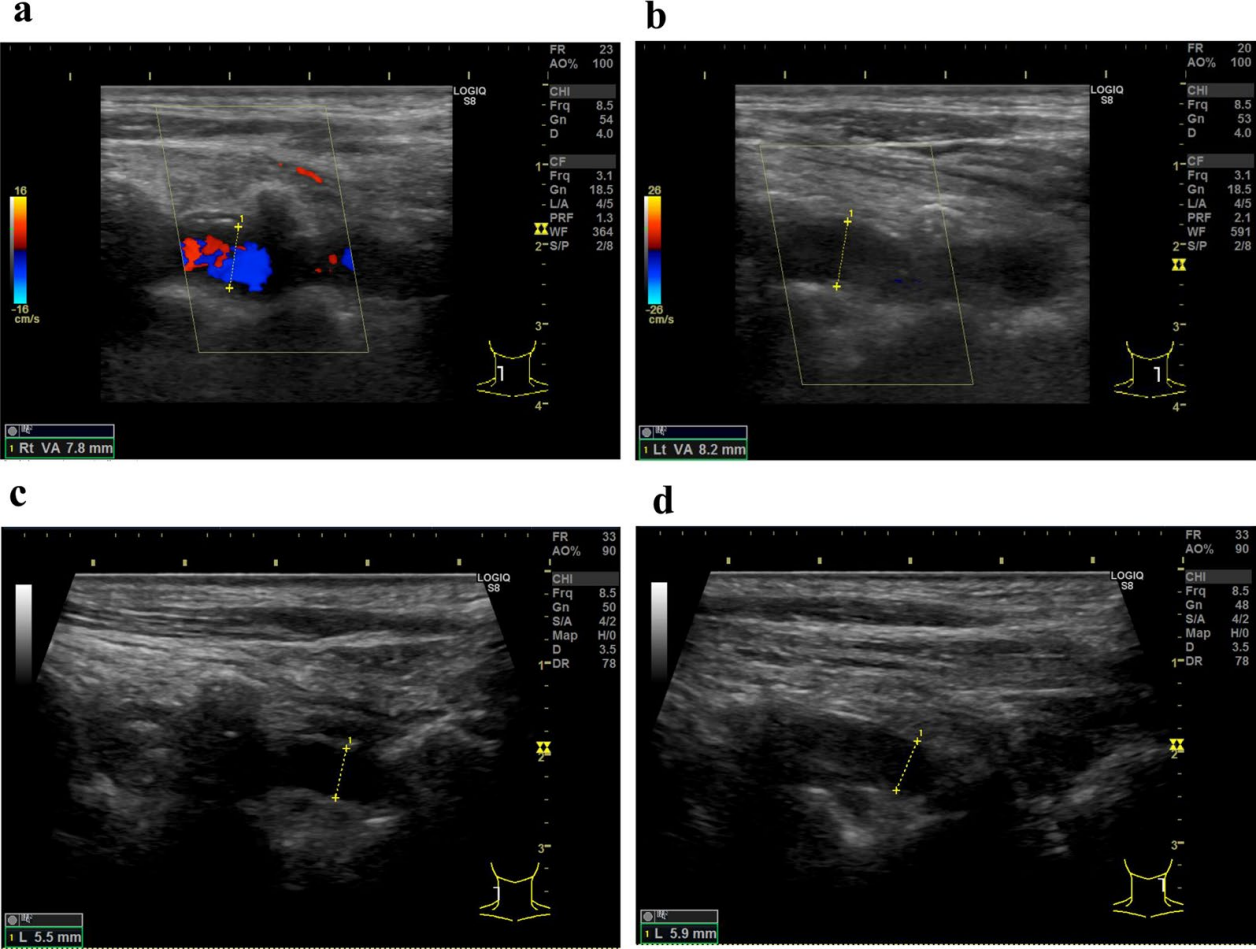
Ultrasound findings of bilateral vertebral arteries before and after treatment. Bilateral vertebral arteries were observed from bifurcation to C3 level by ultrasound. Before treatment, those were dilated to approximately 8 mm on all levels. **a** Right vertebral artery. **b** Left vertebral artery. Four years and 8 months after treatment, the above-mentioned arteries decreased to approximately 5.5 mm on all levels. **c** Right vertebral artery. **d** Left vertebral artery

**Fig. 4:**
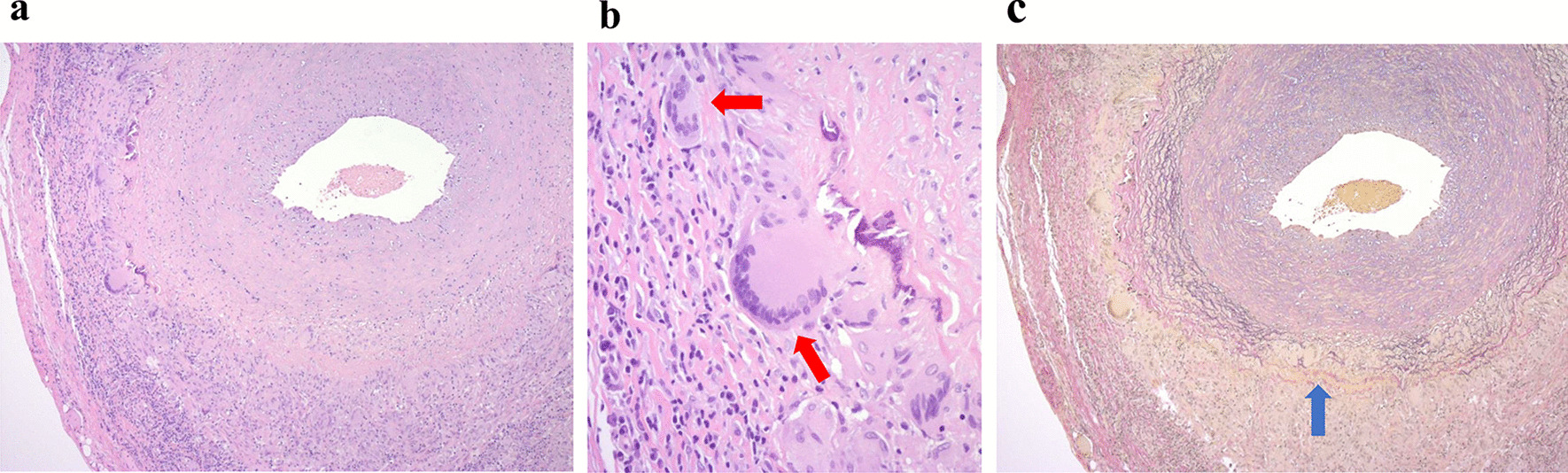
Histopathology of left superficial temporal artery biopsy. **a** and **b** Number of inflammatory cell infiltrates mainly in the media and sporadic multinucleated giant cells (red arrows) with intimal hyperplasia and vascular occlusion (hematoxylin–eosin staining: **a**, ×50; **b**, ×200). **c** Fragmentation of the internal elastic lamina (blue arrow) (Elastica van Gieson staining: **c**, ×50)

A 72-year-old Japanese man with a month-long history of dyspnea on exertion and bilateral shoulder pain gradually presented difficulty in raising both upper limbs. He visited a local doctor and underwent laboratory testing, which revealed an elevated serum C-reactive protein (CRP) (12.62 mg/dL). Hence, he was admitted to our hospital. He was naturally healthy and had no allergies, therefore was not on any medication. He is currently unemployed. His sister has a medical history of rheumatoid arthritis, and his daughter has a medical history of systemic lupus erythematosus. He has smoked 40 cigarettes a day for the past 50 years and does not consume alcohol. On admission, his vital signs were as follows: blood pressure 147/90 mmHg, pulse 110 beats/minute, respiratory rate 12 breaths/minute, and SpO_2_ 98% (room air). He had no headache, jaw claudication, visual loss, or fever. Tenderness in the bilateral temporal arteries was absent, whereas mild tenderness was observed in both shoulders. The upper right limb could not be elevated to shoulder level, whereas the upper left limb could not be elevated at all (Fig. 1a). A physical examination revealed no abnormalities other than those mentioned above. The laboratory tests revealed an increase in CRP and erythrocyte sedimentation rate (ESR) levels (13.99 mg/dL and 31 mm/hour, respectively). Both rheumatoid factor (RF) and anti-cyclic citrullinated peptide antibody (ACPA) were negative, and the matrix metalloproteinase-3 (MMP-3) level remained normal (54.3 ng/mL). Antinuclear antibody and anti-SSA antibody were also negative (Table 1). PMR and EORA were considered as differential diagnoses. However, PMR was unlikely as there were no signs of tenosynovitis and bursitis in both shoulders on musculoskeletal ultrasound examination. Moreover, the total score was 3 points and did not meet the 2012 Provisional Classification Criteria for Polymyalgia Rheumatica (“absence of RF or ACPA”: 2 points, “absence of other joint involvement”: 1 point; Table 2) [7]. EORA was also unlikely because both RF and ACPA were negative, and there were no signs of synovitis on musculoskeletal ultrasound examination. Moreover, the total score was 2 points and did not meet the 2010 Rheumatoid arthritis classification criteria (“joint involvement”: 1 point, “acute-phase reactants”: 1 point; Table 3) [8]. Since he did not complain of pain even if both upper limbs were passively lifted, neurological disease or muscle disease was considered. Precise neurological examination revealed that there was absence of sensory impairment, but deep tendon reflexes were present in bilateral triceps and the lower limbs, except for bilateral biceps and brachioradialis reflexes. Muscle strength was evaluated by conducting a manual muscle test. The muscle strength of the biceps brachii, brachioradialis, supinator teres, deltoid, pectoralis major, supraspinatus, and infraspinatus decreased predominantly on the left side (Table 4). Ultrasonographic examination showed absence of movement in the left diaphragm during breathing. Hence, bilateral C5 and C6 radicular involvement (the ventral root of spinal nerve) and left C4 radicular involvement were suspected. Since cervical magnetic resonance imaging showed no mechanical causality, cervical radiculopathy of unknown origin was suggested. Fluorodeoxyglucose positron emission tomography/computed tomography (^18^F-FDG PET/CT) revealed increased FDG lineal uptake along the vessel walls, including the temporal arteries, vertebral arteries, axillary arteries, descending aorta, and femoral arteries (Fig. 2). Ultrasound examination showed absence of abnormality in the common carotid arteries, internal carotid arteries, and temporal arteries, but bilateral vertebral arteries were dilated to approximately 8 mm from bifurcation to C3 level (Fig. 3a and b). Ultrasound examination did not reveal any abnormality in the temporal artery. However, because ^18^F-FDG PET/CT revealed FDG uptake in the temporal arteries, a left superficial temporal artery biopsy was conducted that showed histopathological findings compatible with GCA (Fig. 4). Thirty milligrams (0.6 mg/kg) of oral prednisolone (PSL) was administered, and CRP test turned negative 10 days later. The patient could almost completely elevate both upper limbs almost immediately, his dyspnea on exertion disappeared, and he was discharged 20 days later. Currently, 4 years and 8 months after treatment, he is healthy and the result of CRP test remains negative with 1 mg of PSL (Fig. 1b) (Additional file 1).

**Table 1:** Laboratory data on admission

Complete blood cell	
White blood cell (/μL)	10,240
Red blood cell (/μL)	3.4 × 10^6^
Hemoglobin (g/dL)	10.4
Platelet (/μL)	79.5 × 10^4^
Coagulation test	
APTT (second)	44.5
PT-INR	1.28
Biochemistry	
HbA1c (%)	6.9
BUN (mg/dL)	18.5
Cr (mg/dL)	0.80
eGFR (mL/minute/1.73 m^2^)	72.57
Na (mEq/L)	137
K (mEq/L)	5.1
Cl (mEq/L)	98
AST (U/L)	78
ALT (U/L)	68
ALP (U/L)	568
LDH (U/L)	359
CK (U/L)	52
T-Bil (mg/dL)	0.47
TP (g/dL)	8.1
Alb (g/dL)	2.3
ESR (mm/hour)	31
Immunochemistry	
CRP (mg/dL)	13.99
IgG (mg/dL)	2744
ANA	< 40
Anti-SS-A Ab (U/mL)	0.6
RF (U/mL)	10.6
ACPA (U/L)	0.5
MMP-3 (ng/mL)	54.3
C3 (mg/dL)	174.1
C4 (mg/dL)	43.8
CH50 (U/mL)	106.7
MPO-ANCA (IU)	< 0.5
PR3-ANCA (IU)	0.6

APTT, activated partial thromboplastin time; PT-INR, prothrombin time–international normalized ratio; HbA1c, hemoglobin A1c; BUN, blood urea nitrogen; Cr, creatinine; eGFR, estimated glomerular filtration rate; Na, sodium; K, potassium; Cl, chlorine; AST, aspartate aminotransferase; ALT, alanine aminotransferase; ALP, alkaline phosphatase; LDH, lactate dehydrogenase; CK, creatine kinase; T-Bil, total bilirubin; TP, total protein; Alb, albumin; ESR, erythrocyte sedimentation rate; CRP, C-reactive protein; IgG, immunoglobulin G; ANA, antinuclear antibody; Anti-SS-A Ab, anti-SS-A antibody; RF, rheumatoid factor; ACPA, anti-cyclic citrullinated peptide antibody; MMP-3, matrix metalloproteinase-3; C3, complement component 3; C4, complement component 4; CH50, 50% hemolytic unit of complement; MPO-ANCA, myeloperoxidase-anti-neutrophil cytoplasmic antibody; PR3-ANCA, proteinase-3-anti-neutrophil cytoplasmic antibody

**Table 2:** Polymyalgia rheumatica classification criteria scoring algorithm—required criteria: age ≥ 50 years, bilateral shoulder aching, abnormal C-reactive protein level, and/or erythrocyte sedimentation rate

	Points without US (0–6)	Points with US (0–8)
Morning stiffness duration **>** 45 minutes	2	2
Hip pain or limited range of motion	1	1
Absence of RF or ACPA	2	2
Absence of other joint involvements	1	1
At least one shoulder with subdeltoid bursitis and/or biceps tenosynovitis and/or glenohumeral synovitis (either posterior or axillary), and at least one hip with synovitis and/or trochanteric bursitis	NA	1
Both shoulders with subdeltoid bursitis, biceps tenosynovitis, or glenohumeral synovitis	NA	1

An individual with a score of ≥ 4 is categorized as having PMR in the algorithm without US, and an individual with a score of ≥ 5 is categorized as having PMR in the algorithm with US

PMR, polymyalgia rheumatica; CRP, C-reactive protein; ESR, erythrocyte sedimentation rate; US, ultrasonography; RF, rheumatoid factor; ACPA, anti-cyclic citrullinated peptide antibody; NA, not applicable

**Table 3:** The 2010 American College of Rheumatology/European League Against Rheumatism classification criteria for rheumatoid arthritis

	Score
Target population (who should be tested?): patients who	
1) have at least one joint with definite clinical synovitis (swelling)^a^	
2) have synovitis that is not attributable to another disease^b^	
Classification criteria for RA (score-based algorithm: add the scores in categories A–D; a score of ≥ 6/10 is needed for the classification of a patient as having definite RA)^c^	
(A) Joint involvement^d^	
1 large joint^e^	0
2−10 large joints	1
1−3 small joints (with or without involvement of large joints)^f^	2
4−10 small joints (with or without involvement of large joints)	3
> 10 joints (at least one small joint)^g^	5
(B) Serology (at least one test result is needed for classification)^h^	
Negative RF and ACPA	0
Low-positive RF or ACPA	2
High-positive RF or ACPA	3
(C) Acute-phase reactants (at least one test result is needed for classification)^i^	
Normal CRP level and ESR	0
Abnormal CRP level or normal ESR	1
(D) Duration of symptoms^j^	
< 6 weeks	0
≥ 6 weeks	1

^a^The criteria are established for the classification of patients newly presenting with symptoms. In addition, patients with erosive disease typical of RA with a history compatible with prior fulfillment of the 2010 criteria should be classified as having RA. Patients with long-standing disease, including those whose disease is inactive (with or without treatment), and who, based on retrospectively available data, have previously fulfilled the 2010 criteria should be classified as having RA

^b^Differential diagnoses differ in patients with different presentations but may include conditions such as systemic lupus erythematosus, psoriatic arthritis, and gout. If the relevant differential diagnoses to consider are unclear, an expert rheumatologist should be consulted

^c^Although patients with a score of < 6/10 are not classifiable as having RA, their status can be reassessed, and the criteria might be fulfilled cumulatively over time

^d^Joint involvement refers to any swollen or tender joint on examination, which may be confirmed by imaging evidence of synovitis. The distal interphalangeal, first carpometacarpal, and first metatarsophalangeal joints are excluded from the assessment. Categories of joint distribution are classified according to the location and number of involved joints, with placement into the highest category possible based on the pattern of joint involvement

^e^“Large joints” refers to the shoulders, elbows, hips, knees, and ankles

^f^“Small joints” refers to the metacarpophalangeal, proximal interphalangeal, second to fifth metatarsophalangeal, and thumb interphalangeal joints, as well as the wrists

^g^In this category, at least one of the involved joints must be a small joint; the other joints can include any combination of large and additional small joints and other joints not specifically listed elsewhere (for example, temporomandibular, acromioclavicular, and sternoclavicular)

^h^“negative” refers to international unit values that are less than or equal to the upper limit of normal for the laboratory test and assay; “low-positive” refers to international unit values that are higher than the limit of normal but ≤ 3 times the limit of normal for the laboratory test and assay; and “high-positive” refers to international unit values that are more than three times the limit of normal for the laboratory test and assay. When RF information is only available as “positive” or “negative,” a “positive” result should be scored as “low-positive” for RF

^i^Normal/abnormal is determined on the basis of local laboratory standards

^j^“Duration of symptoms” refers to patients’ self-report of the duration of signs or symptoms of synovitis (for example, pain, swelling, and tenderness) of the joints that are clinically involved at the time of assessment, regardless of the treatment status

RA, rheumatoid arthritis; RF, rheumatoid factor; ACPA, anti-citrullinated protein antibody; CRP, C-reactive protein; ESR, erythrocyte sedimentation rate

**Table 4:** Manual muscle test on admission

	Right	Left	Dominant nerve
Biceps brachii	3/5	1/5	C5–C6
Triceps brachii	5/5	5/5	C6–C7–C8
Brachioradialis	5/5	1/5	C5–C6
Supinator teres	5/5	1/5	C5–C6
Pronator teres	5/5	5/5	C6–C7
Deltoid	3/5	1/5	C5–C6
Pectoralis major	3/5	3/5	C5–C6–C7–C8–T1
Trapezius	5/5	5/5	C3–C4
Supraspinatus	3/5	1/5	C5–C6
Infraspinatus	3/5	*	C5–C6
Rhomboids	5/5	5/5	C4–C5
Latissimus dorsi	5/5	5/5	C6–C7–C8

C, cervical; T, thoracic

^*^We could not assess the patient’s left infraspinatus muscle strength as he was unable to get the correct limb position

## Discussion and conclusions

We reported a case of GCA with cervical radiculopathy presenting clinical symptoms similar to those of PMR or EORA.

PMR is an inflammatory disorder that occurs in elderly people older than 50 years. PMR is characterized by pain in both shoulders, neck, and hip girdle, and morning stiffness of more than 45 minutes. Moreover, nonspecific symptoms such as general fatigue and malaise may be observed. Elevated serum inflammatory markers are a typical feature [7, 9].

EORA is also an inflammatory disease that occurs in elderly people older than 60 years, and it is prevalent in men, has acute onset, involves the large and proximal joints, and has less RF positivity [10, 11]. In this case, although PMR and EORA were initially suspected because of old age, pain in both shoulders, difficulty in raising both upper limbs, and increased inflammatory response, the possibility of the incidence of these diseases was unlikely based on musculoskeletal ultrasound findings and each classification criteria. A precise neurological examination suggested cervical radiculopathy of unknown origin, and subsequent ^18^F-FDG PET/CT findings suggested GCA. The biopsy of the left superficial temporal artery confirmed the diagnosis of GCA.

Recently, a characteristic ^18^F-FDG PET/CT finding of PMR has been reported; FDG uptake was observed not only in the shoulder and hip joints but also in the ischial tuberosity, spinous process, and so on [9, 12–14]. In this case, the lack of uptake of FDG in the ischial tuberosity and spinous process did not indicate PMR. Similarly, because FDG was not taken up in the joints at all, EORA could be ruled out.

As the mechanism of radiculopathy in GCA, vasculitis of the vertebral arteries could reduce blood flow in the radicular arteries, which supply blood to the spinal nerve roots [15]. Moreover, prior autopsy reports confirmed vasculitis in the radicular arteries rather than in the proximal feeding vessels [16].

In this case, radiculopathy occurred mainly in the C5 nerve as previously reported [5, 6]. The following mechanism has been proposed to explain C5 nerve dominance. The radicular arteries at the C1–C6 levels receive blood supply from the vertebral arteries, whereas the radicular arteries at the C7–T1 levels receive blood supply from the thyrocervical and costocervical trunks. Therefore, even if vertebral artery vasculitis occurs, the blood flow is relatively maintained at the C7–T1 levels, and radiculopathy is less likely to occur. The radicular arteries at the C1–C4 levels are also compensated by blood flow from the anterior spinal artery; hence, the C5 and C6 nerve roots are susceptible to radiculopathy [17].

It has also been reported that the muscles that control the C1–C3 nerves are located in the head and neck, and even if radiculopathy occurs in that area, it is difficult to establish a diagnosis [5]. Andrzejczak *et al*. reported the sources of vascularization of the brachial plexus using six autopsy cadavers. The vertebral and ascending cervical arteries were the most common sources of blood flow to the C5 and C6 nerve roots (both 35.29%). The subclavian artery predominantly supplied blood flow to the C7 nerve roots (66.67%) and the vertebral artery (8.33%). The deep cervical artery was the predominant source of blood flow to the C8 and T1 nerve roots (64.71%), but the vertebral artery is not a source of blood flow (0%). Therefore, C5 and C6 radiculopathy may be more likely to occur if vertebral artery vasculitis occurs; the blood vessels supplying the C1–C4 nerve roots are not described [18].

In this case, both upper limbs can be raised relatively immediately after treatment, and the treatment response is very similar to that of PMR. If radiculopathy occurs only due to ischemia as previously reported, the mechanism behind how both upper limbs can be elevated immediately after treatment remains unknown.

In this case, the vertebral arteries were among the affected blood vessels, and patient’s ultrasound revealed that the luminal diameter of bilateral vertebral arteries was dilated to approximately 8 mm before treatment and improved to approximately 5.5 mm 4 years and 8 months after treatment (Fig. 3). The luminal diameter of the vertebral artery was reported to be 3.6 ± 0.4 mm (mean ± SD) in healthy adults aged 60–85 years on ultrasound [19]. Anatomically, the vertebral arteries generally enter the transverse foramen at the C6 level and ascend, and the vertebral arteries and the cervical nerve roots are very close to each other at the C1–C6 levels. Therefore, the spread of inflammation of the vertebral artery can cause radiculopathy; once the inflammation improves after steroid treatment, the weakness in the upper limbs will also improve. However, this hypothesis is only speculative and needs to be evaluated in the future.

If this case was misdiagnosed as PMR or EORA based only on the clinical symptoms and laboratory data, the patient is expected to demonstrate CRP positivity, difficulty in raising both upper limbs, and dyspnea on exertion due to insufficient steroid treatment. If the patient continues to receive inadequate treatment, vascular complications may occur later. Therefore, it is essential to recognize that peripheral neuropathy of GCA can be manifested with radicular symptoms commonly affecting C5 nerve root. If the typical clinical symptoms of GCA are absent, then the clinical symptoms are similar to those of PMR or EORA. The musculoskeletal ultrasound and precise neurological examination were the turning points for the diagnosis of this case, and making a careful diagnosis using these methods was thought to be important.

## Supplementary Information

**Additional file 1:** CARE flow diagram. Initial and Follow-up Patient Visit Documentation.

## Data Availability

Not applicable.

## Abbreviations

GCA: Giant cell arteritis
PMR: Polymyalgia rheumatica
EORA: Elderly-onset rheumatoid arthritis
CRP: C-reactive protein
ESR: Erythrocyte sedimentation rate
RF: Rheumatoid factor
ACPA: Anti-cyclic citrullinated peptide antibody
MMP-3: Matrix metalloproteinase-3
^18^F-FDG PET/CT: Fluorodeoxyglucose positron emission tomography/computed tomography
PSL: Prednisolone
