# Supplementary figures and images for: Giant cell arteritis with cervical radiculopathy mimicking polymyalgia rheumatica and elderly-onset rheumatoid arthritis: a case report
Source: J Med Case Rep. 2021 Oct 20;15:527. doi: 10.1186/s13256-021-03107-7 (PMC8527715; doi:10.1186/s13256-021-03107-7)

## Flow Diagram — Case Reports following the CARE guidelines

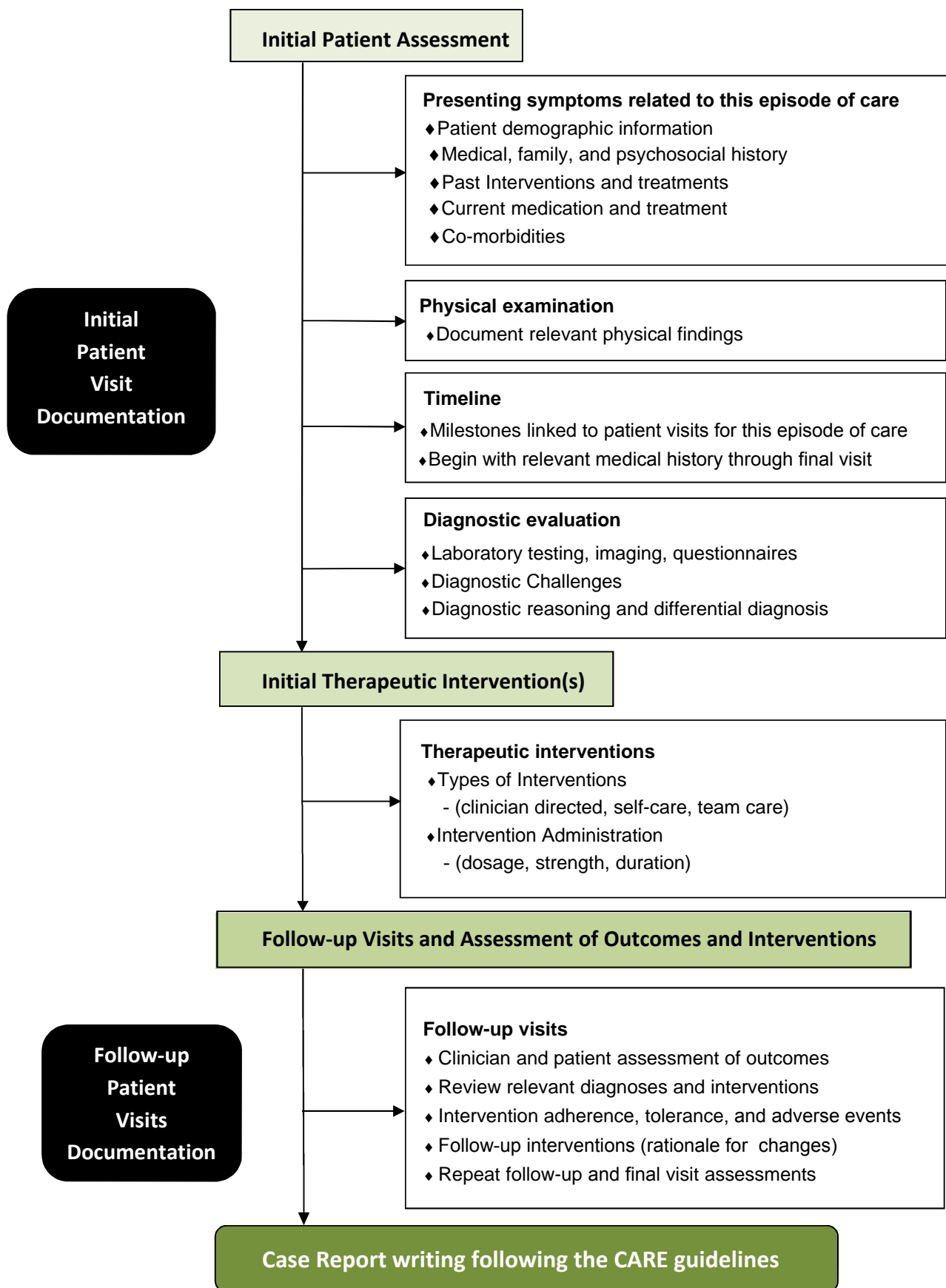

Supplement: Supplementary file 1 — Additional file 1: CARE flow diagram. Initial and Follow-up Patient Visit Documentation. [file 13256_2021_3107_MOESM1_ESM.pdf]
